# The role of Cas8 in type I CRISPR interference

**DOI:** 10.1042/BSR20150043

**Published:** 2015-06-11

**Authors:** Simon D.B. Cass, Karina A. Haas, Britta Stoll, Omer S. Alkhnbashi, Kundan Sharma, Henning Urlaub, Rolf Backofen, Anita Marchfelder, Edward L. Bolt

**Affiliations:** * School of Life Sciences, University of Nottingham, Queen's Medical Centre, Nottingham NG7 2UH, U.K.; † Biology II, Ulm University, 89069 Ulm, Germany; ‡ Bioinformatics group, Department of Computer Science, University of Freiburg, Georges-Köhler-Allee 106, 79110 Freiburg, Germany; § Max Planck Institute of Biophysical Chemistry, Am Faßberg 11, 37077 Göttingen, Germany; ║ Bioanalytics Research Group, Institute of Clinical Chemistry, University Medical Center Göttingen, Göttingen, Germany; ¶ Centre for Biological Signalling Studies (BIOSS), Cluster of Excellence, University of Freiburg, Germany

**Keywords:** archaea, CRISPR-associated (Cas)8, CRISPR-associated complex for antiviral defence (Cascade), clustered regularly interspaced short palindromic repeat (CRISPR), nuclease

## Abstract

CRISPR (clustered regularly interspaced short palindromic repeat) systems provide bacteria and archaea with adaptive immunity to repel invasive genetic elements. Type I systems use ‘cascade’ [CRISPR-associated (Cas) complex for antiviral defence] ribonucleoprotein complexes to target invader DNA, by base pairing CRISPR RNA (crRNA) to protospacers. Cascade identifies PAMs (protospacer adjacent motifs) on invader DNA, triggering R-loop formation and subsequent DNA degradation by Cas3. Cas8 is a candidate PAM recognition factor in some cascades. We analysed Cas8 homologues from type IB CRISPR systems in archaea *Haloferax volcanii* (Hvo) and *Methanothermobacter thermautotrophicus* (Mth). Cas8 was essential for CRISPR interference in Hvo and purified Mth Cas8 protein responded to PAM sequence when binding to nucleic acids. Cas8 interacted physically with Cas5–Cas7–crRNA complex, stimulating binding to PAM containing substrates. Mutation of conserved Cas8 amino acid residues abolished interference *in vivo* and altered catalytic activity of Cas8 protein *in vitro*. This is experimental evidence that Cas8 is important for targeting Cascade to invader DNA.

## Abstract (precis)

We have used genetic and protein biochemical analyses in two archaeal species to identify that Cas8 is essential for CRISPR interference. We provide evidence that Cas8 functions as part of archaeal Cascade, an R-loop forming nucleoprotein complex, recognizing protospacer adjacent motifs (PAMs) on invader DNA. An RNA nuclease activity of Cas8 is also described.

## INTRODUCTION

CRISPR (clustered regularly interspaced short palindromic repeat) systems were discovered in *Streptococcus thermophilus* [1], by providing adaptive immunity to invasive genetic elements, recently reviewed in [2,3]. Immunity arises from base pairing of host encoded CRISPR RNA (‘crRNA’) with invader DNA/RNA, promoting nucleolytic degradation of the invader, processes called ‘interference’. DNA sequences that are targeted by crRNA are called ‘protospacers’ and can be identified from an archive of previously encountered protospacers arrayed in a CRISPR locus as ‘spacers’, separated from one another by repeat sequences. ‘Adaptation’ or ‘spacer acquisition’ processes furnish CRISPR with new spacer-repeat units requiring two highly conserved CRISPR-associated (‘Cas’) proteins, Cas1 and Cas2. Cas1–Cas2 adaptation may be functionally linked to interference (‘primed’) or not (‘naïve’), in each case by mechanisms unclear, reviewed in [3–5].

Cas proteins that catalyse interference show substantial diversity, with current classification into three major groups, types I, II and III [6–8], characterized by distinct effector complexes that manoeuvre crRNA to base pair with target DNA or RNA. Type I, II and IIIA systems target DNA catalysed by respectively, ‘Cascade’ (Cas complex for antiviral defence) [9,10], Cas9 [11,12] or CSM complex [13,14]. In contrast, CMR complexes target RNA in type IIIB CRISPR systems [15–17].

Cascades catalyse interference in type I CRISPR immunity. They are nucleoprotein assemblies of crRNA and Cas proteins (To help follow the variable nomenclature used in the literature for Cas proteins within Cascade complexes: CasA=Cse1/Cas8; CasC=Cas7; CasD=Cas5; CasE=Cas6e.). Diversity of amino acid sequence and gene synteny between Cascade components prompted categorization of type I CRISPR systems into sub-types A–F [6], with further refinement into types A–G to accommodate variants in types IA, ID and IG [18]. Common structural features are observed in Cascades from the different CRISPR sub-types in bacteria and archaea [9,10,19–22]. crRNA is delivered into Cascade as a single spacer element sequence after truncation of crRNA transcripts by Cas5 and Cas6 nucleases [10,19,20,23–28], reviewed in [29,30]. *Sulfolobus solfataricus* [19] and *Thermoproteus tenax* [21] Cascades (both type IA) and *Escherichia coli* (type IE) [10] use multiple copies of Cas7 protein to form a backbone filament with crRNA, functionally analogous to the Csy3-crRNA backbone of *Pseudomonas aeruginosa* Cascade (Type IF) [20]. Another variation of Cascade subunit type with common function is observed in type IC systems that use Csd2 to form Cas7-like crRNA filaments [31]. Interference is established by Cascade base pairing crRNA with protospacer (invader) DNA through ‘seeding’ [20,32–35] and further into an R-loop [9], reviewed in [36] that promotes nucleolytic degradation of invader DNA probably by interaction of Cascade with Cas3 helicase-nuclease. Recent atomic structures of *E. coli* Cascade complex have provided detailed insight into the arrangement of protein subunits relative to one another and to crRNA to provide a mechanism for interference in type IE CRISPR [37–39].

Cascade can identify invader DNA by interaction with PAM (protospacer adjacent motif) sequences. PAMs are short (2–5 nt) sequences located on invader DNA upstream of the protospacer that trigger Cascade–Cas3 interference [40]. A mechanism for Cascade–PAM recognition described in *E. coli* involves a CasA ‘large subunit’ contacting target DNA via its ‘L1 loop’ [41,42], as part of multiple interactions with Cas5 [37–39]. CasA is the ‘signature’ protein of type IE CRISPR systems, essential for Cascade function [6]. Atomic resolution structures of Cascade [37–39] show CasA interlocking with CasD, contributing to binding of the crRNA 5′-handle. CasA also contacts PAM as part of the tight association with CasD. In other type I CRISPR systems Cas8 is predicted to be functionally analogous to CasA [18], as a ‘signature’ protein for type IA, IB and IC systems, referred to respectively, as Cas8a2, Cas8b and Cas8c [6]. A more recent analysis highlighted diversity of Cas8 proteins leading to their renaming as Cas8, Cas8′ and Cas8″ proteins and creating new type-I CRISPR variants based on Cas8 protein sequences and positioning of *cas8* genes relative to other *cas* genes [18]. An important role for Cas8 in interference was previously demonstrated in the euryarchaeon *Haloferax volcanii* (Hvo) [43]. Here we report genetic and biochemical analyses of Cas8 homologues from Hvo and *Methanothermobacter thermautotrophicus* (Mth).

### EXPERIMENTAL

### Cultivation of *Haloferax volcanii* strains

*H. volcanii* strains H119 (Δ*leuB*, Δ*pyrE2*, Δ*trpA*) [44] and Δ*cas8* [22] were grown aerobically at 45°C in Hv-YPC medium [45]. *H. volcanii* strains Δ*cas8* containing plasmids with mutated *cas8* genes were cultivated in Hv-Ca medium supplemented with 0.25 mM tryptophan (Trp). *E. coli* strains DH5α (Invitrogen) and GM121 were grown aerobically at 37°C in 2YT medium [46].

### Transformation of *H. volcanii* and generation of strain Δ*cas8*

For transformation of *H. volcanii*, plasmids were passaged through methylase deficient *E. coli* GM121 cells and introduced into *H. volcanii* by the PEG method [44]. Transformants were plated on selective media. Gene deletion in *H. volcanii* was performed as described previously [47]. Briefly, an integrative plasmid carrying flanking regions of the gene to be deleted and the *pyr*E2 gene as an auxotrophic marker, was incorporated into the genome by homologous recombination. Removal of this plasmid was forced by supplementing the media using 5-fluoroorotic acid (5-FOA, final concentration 50 μg/ml) that is converted to toxic 5-fluorouracil by orotate phosphoribosyltransferase encoded by *pyr*E2 gene. Positive clones were selected by colony PCR and gene deletion was subsequently confirmed by Southern blot hybridization. Using this method *cas8* was deleted resulting in strain Δ*cas8*.

### Plasmids for *Haloferax volcanii*

Primers and plasmids are detailed in Supplementary Information. For generation of the integrative plasmid for *cas8* deletion, a *cas8* fragment with up- and downstream flanking regions (546 and 501 bp respectively) was amplified from genomic DNA by PCR using the oligonucleotides Csh1KOup, Csh1KOdo and Phusion DNA polymerase (Biozym). This fragment was ligated in pTA131 digested with EcoRV. With this plasmid, an inverse PCR was performed using the oligonucleotides IPCsh1KOup and IPCsh1KOdo, followed by ligation of the PCR product to obtain the integrative plasmid pTA131-*cas8*updo with *cas8* flanking regions only [48].

For complementation of *cas8*, pTA927-N-FLAG-*cas6* [22] was digested with HindIII and BamHI to remove *cas6* and to subsequently insert the *cas8* gene. The insert was generated by a PCR on genomic DNA with oligonucleotides 5-HindIII-cas8 and 3-cas8-NcoI-BamHI and subsequent digestion of the PCR product with HindIII and BamHI. Mutations were introduced into the *cas8* gene using the QuikChange® II-Site Directed Mutagenesis Kit (Agilent Technologies). Both pTA927-N-FLAG-*cas8* and pTA927-*cas8*-mutX were used for complementation of deletion strain Δ*cas8*. For interference tests, the four PAM sequences that have been identified for *H. volcanii* TAA (PAM25), TAT (PAM26), TAG (PAM27) and CAC (PAM54) [43] were subcloned in pTA352 [49] together with sequence of spacer 1 from locus P1. Therefore, pTA409-PAM25, pTA409-PAM26, pTA409-PAM27 and pTA409-PAM54 as well as pTA352 (without any insert) were digested each with XhoI and BamHI according to previous studies [35] and the PAM-spacer fragments were ligated each with pTA352 to obtain pTA352-PAM25, pTA352-PAM26, pTA352-PAM27 and pTA352-PAM54.

### Northern blot analysis of crRNA formation in *Haloferax volcanii*

From *H. volcanii* cultures grown to exponential growth phase, total RNA was isolated using TRIzol® Reagent (Life Technologies) and remaining DNA was digested with RQ1 RNase-Free DNase (Promega). Ten micrograms RNA was separated on 8% urea-polyacrylamide gels and transferred to a nylon membrane (Hybond-N+, GE Healthcare). For detection of crRNA, the oligonucleotide probes against spacer 1 from locus P1 and 5S RNA (control) were labelled radioactively using γ-^32^P-ATP and T4 polynucleotide kinase (Thermo Scientific). Signals were detected with a radiosensitive photofilm (GE Healthcare).

### Interference tests in *Haloferax volcanii*

A plasmid based invader assay [43] was performed to test functionality of Δ*cas8* x pTA927-N-FLAG-*cas8* and Δ*cas8* x pTA927-*cas8*-mutX in the interference reaction. Plasmid invaders pTA352-PAM3 [43], pTA352-PAM9 [43], pTA352-PAM25 (present study), pTA352-PAM26 (present study), pTA352-PAM27 (present study) and pTA352-PAM54 (present study) were used and the vector pTA352 (without any insert) served as control. Transformants were plated on Hv-Min+Trp medium without leucine and uracil. Interference tests were performed at least three times to obtain statistically relevant data and activity in interference was defined for minimum 100-fold reduction in transformation rate.

### Co-purification of Cas proteins with N-FLAG-Cas7 and identification by MS

H119 was transformed with pTA927-N-FLAG-*cas7* and cells grown to a *D*_650_ of 0.6 in medium containing 0.25 mM Trp to induce protein expression. To further induce protein expression additional Trp was added to a final concentration of 3 mM. The culture was incubated for further 3 h, cells were pelleted and washed once with salt-enriched PBS buffer [2.5 M NaCl, 150 mM MgCl_2_, 1 × PBS (137 mM NaCl, 2.7 mM KCl, 8 mM Na_2_HPO_4_, 2 mM K_2_HPO_4_, pH 7.4)]. Cells were resuspended in lysis buffer [100 mM Tris/HCl, pH 7.5, 10 mM EDTA, 10 mM MgCl_2_, 1 mM CaCl_2_, 8 units/μl DNase RQ1 (Promega), 13 μl/ml protease inhibitor cocktail (Sigma)], incubated for 30 min at 4°C and subsequently lysed by sonication. Cell lysate clarified by ultracentrifugation (15 min at 100,000 ***g***) and 0.03 volume of 5 M NaCl was added to the resulting supernatant. For subsequent FLAG-affinity purification, the supernatant was incubated over night at 4°C with anti-FLAG M2 affinity gel (Sigma) equilibrated with precooled washing buffer (0.2 M Tris/HCl, pH 7.4, 0.5 M NaCl). After washing, FLAG-tagged Cas7 was eluted by adding 3× FLAG peptide (150 ng/μl in washing buffer; Sigma). Proteins of the elution fraction were separated by SDS/PAGE (8% polyacrylamide) which was subsequently stained with Coomassie. The proteins were in-gel digested with trypsin as described in [50]. Peptides extracted from the in-gel digestion were analysed by LC–MS/MS on an Orbitrap XL instrument (Thermo Fischer Scientific) under standard conditions. The fragment spectra obtained for peptides were searched against *H. volcanii* database (www.halolex.mpg.de) [51] using MASCOT as a search engine. Peptides with the peptide score lower than 20 were considered unspecific.

### *Methanothermobacter* Cas8′ cloning, gene expression and protein purification

DNA primer sequences for cloning and mutagenesis are listed in Supplementary Material. Cas8′ (ORF Mth 1090) was amplified by PCR from *M. thermautotrophicus* ΔH genomic DNA. The gene fragment cloned into pET14b facilitated expression of N-terminal hexahistidine-tagged Mth Cas8′ (His_6_–Cas8′). Site-directed mutagenesis of Cas8′ was based on the Quick-change protocol, with mutations verified by DNA sequencing. His_6_–Cas8′ protein was expressed in *Escherichia coli* strain BL21 Codon Plus, at 37°C with expression induced by IPTG (0.5 mM) at *D*_600_ 0.6 for 2–4 h at 30°C. Harvested cells were resuspended in buffer B (20 mM Tris/HCl, pH 8.0, 500 mM NaCl, 5 mM imidazole) containing PMSF (0.1 mM) and freeze-thawed prior to lysis by sonication, followed by centrifugation at 39000 ***g*** for 20 min. His_6_–Cas8′ was purified on an AKTA–FPLC with each step followed by SDS/PAGE. Soluble proteins were loaded on to a 5 ml of His Trap FF column charged with nickel chloride and equilibrated in buffer B. His_6_–Cas8′ eluted into fractions within a gradient of 5—500 mM imidazole in buffer B were pooled and loaded on to a HI Load Superdex 200 26/60 column equilibrated in buffer C (20 mM Tris/HCl, pH 8.0, 150 mM NaCl, 1 mM DTT and 0.1mM PMSF) followed by elution in the same buffer in one column volume. His_6_–Cas8′ fractions were pooled and loaded on to 5 ml of heparin HP column equilibrated in buffer C. His_6_–Cas8′ eluted in a gradient of 150–1500 mM NaCl and fractions containing His_6_–Cas8′ were pooled and dialysed into buffer D [20 mM Tris/HCl, pH 8.0, 500 mM KoAc, 1 mM DTT, 0.1 mM PMSF and 40% (w/v) glycerol] for storage in aliquots at −80°C. Mutant Cas8′ proteins were purified using the same methods.

### Nucleic acid substrates for analysis of Cas8′ *in vitro*

Oligonucleotides were purchased from MWG and are listed in Supplementary Materials. Labelling of oligonucleotides and their annealing into substrates followed standard methods, summarized briefly: oligonucleotide (300 ng) was 5′-end-labelled with ^32^P from γ^32^P-ATP using T4 polynucleotide kinase (NEB). Labelled oligonucleotide was purified from unincorporated γ^32^P-ATP in BioSpin6 columns (Bio-Rad) followed by annealing of appropriate oligonucleotide mixtures in sodium citrate buffer. Substrates were purified by electrophoresis through 10% polyacrylamide/1× Tris-Borate-EDTA (TBE) electrophoresis, for 3 h at 120 V and then excision of the appropriate band, detected on photographic film and elution of DNA by diffusion into Tris (10 mM)–NaCl (50 mM) buffer, pH 7.5, at 4°C.

### EMSA

EMSAs mixed protein(s) with substrate in buffer HB [100 mM Tris/HCl, pH 7.5, 10 mM DTT, 500 μg/ml BSA and 30% (v/v) glycerol], typically incubated at 44.8°C for 10 min. Reactions were then mixed by pipetting and loaded directly into wells of a gel comprising 7% polyacrylamide in 1× TBE buffer. Protein–nucleic acid complexes were separated by electrophoresis at 105 V for approximately 170 min in 1× TBE running buffer and detected by gel drying and phosphorimaging. Protein–nucleic acid complex formation was quantified compared with a no-protein control, using AIDA software to calculate the percentage of substrate bound and plotting in Prism to determine binding affinity expressed as *K*_D_.

### Nuclease assays

His_6_–Cas8′ proteins were mixed with substrates (2 nM) in HB buffer supplemented with either 10 mM MgCl_2_, 5 mM EDTA or nothing and incubated at 44.8°C for 10 min. Reactions were terminated by addition of 3 μl of stop solution [2.5% (w/v) SDS, 200 mM EDTA and 10 mg/ml proteinase K] and loaded into 10% TBE non-denaturing gels or 15% polyacrylamide/urea denaturing gels. Gels were dried, imaged and analysed as for EMSAs.

### Protein–protein interactions

The gene encoding *cas5* (ORF Mth1087) was amplified from *M. thermautotrophicus* (Mth) ΔH genomic DNA by PCR and the gene fragment cloned into pMal-C2x for expression of Mth Cas5 fused at its N-terminus to *E. coli* maltose-binding protein (MBP–Cas5). MBP tagging of Mth Cas5 greatly improved its solubility and stability for expression in *E. coli*. *cas7* (ORF Mth1088) was amplified similarly to *cas5*, for cloning into pCDF-1b generating a non-tagged Cas7 protein. Co-expression of MBP-Cas5 and Cas7 in *E. coli* strain BL21 Codon Plus was in broth containing additional glucose (0.2% w/v), protein expression being induced by addition of IPTG (1 mM) at *D*_600_ between 0.4–0.5. Cas5–Cas7 was purified as a complex through multiple steps on an AKTA–FPLC, followed using SDS/PAGE. Clarified soluble proteins were loaded into a column containing 5 ml of amylose sepharose resin and equilibrated in amylose column-binding buffer (ACBB; 20mM Tris/HCl, pH 8.0, 100 mM NaCl, 1 mm DTT and 0.1 mM PMSF). MBP–Cas5 and Cas7 co-eluted within a gradient of 0–5 mM maltose in ACBB and fractions containing MBP–Cas5–Cas7 were pooled and loaded on to 5 ml of Heparin HP column equilibrated in buffer C. MBP–Cas5–Cas7 co-eluted in a gradient of 150–1500 mM NaCl and fractions containing MBP–Cas5–Cas7 were pooled and dialysed into buffer D [20 mM Tris/HCl, pH 8.0, 500 mM KoAc, 1 mM DTT and 40% (w/v) glycerol] for storage in aliquots at −80°C.

MBP–Cas5–Cas7 was used to test for physical interaction with Cas8′. Fifty microlitres of amylose resin slurry was equilibrated in 100 μl of wash buffer (W; 20 mM Tris/HCl, pH 8.0, 150 mM NaCl, 1 mM EDTA and 1% Tween) and centrifuged at 700 ***g*** for 30 s, supernatant removed and washing repeated five times. Twenty micrograms of MBP–Cas5–Cas7, His_6_–Cas8′ or MBP–Cas5 and–Cas7 and His_6_–Cas8b′ we added to the resin to a final volume of 500 μl and end-to-end mixed for 2–4 h at 4°C. Resin was pelleted as before and washed three times as previously. SDS/PAGE disruption buffer was added to resin pellet and boiled. First wash and pellet analysed via SDS/PAGE. Two identically loaded SDS/PAGE gels were used for electroblotting on to PVDF and western blotting to detect the presence of MBP–Cas5 or His_6_–Cas8′ proteins via their affinity tags. Membranes were incubated overnight at 4°C in western blocking buffer (WBB; 50 mM Tris/HCl, pH 7.6, 150 mM NaCl and 0.1% Tween, supplemented with 5% milk powder), before probing each separately with monoclonal antibodies against MBP (NEB) or His_6_ (Sigma). Washed membranes were then probed with HRP-conjugated anti-mouse antibody (against His_6_) or anti-goat antibody (against MBP) to develop using an ECL detection kit and imaged using FujiFilm LAS300 machine.

## RESULTS

### Mutations in Cas8 that inactivate CRISPR interference

Plasmid protection assays in *H. volcanii* (Hvo) identified multiple PAM sequences and that disruption of *cas8* abolished interference [43]. To investigate Cas8 further, Δ*cas8* Hvo cells were analysed in plasmid protection assays when expressing mutant or wild-type Cas8 from a second plasmid (Figure 1A). Single amino acid substitutions were introduced into Hvo Cas8 based on the alignment with Cas8 homologues from archaea and a bacterium (summarized in Figures 1B and 1C and a full alignment in Supplementary Figure S1). Cas8 proteins are diverse [18] with low overall sequence identity, but conserved amino acids were identified to investigate Cas8 function (Figure 1C). Mutation in Hvo Cas8 Asp^230^, Asn^625^ and Leu^627^ abolished interference, equivalent to cells lacking *cas8* (Figure 1D). Mutated Asn^232^ showed reduced interference, by ~20%, toward plasmids with PAM 5′-TTC, but had little effect on interference toward plasmid with PAM 5′-ACT (Figure 1D). Additional assays on Δ*cas8* Hvo pN232A-Cas8 using six Hvo PAMs highlighted a PAM bias, with interference reduced when PAM 5′-TTC or 5′-CAC was used, but with no effect of N232A on the other PAMs (Figure 1E). These genetic assays identified regions of Cas8 that are essential for interference and suggest that Cas8 is sensitive to PAM sequences.

**Figure 1:**
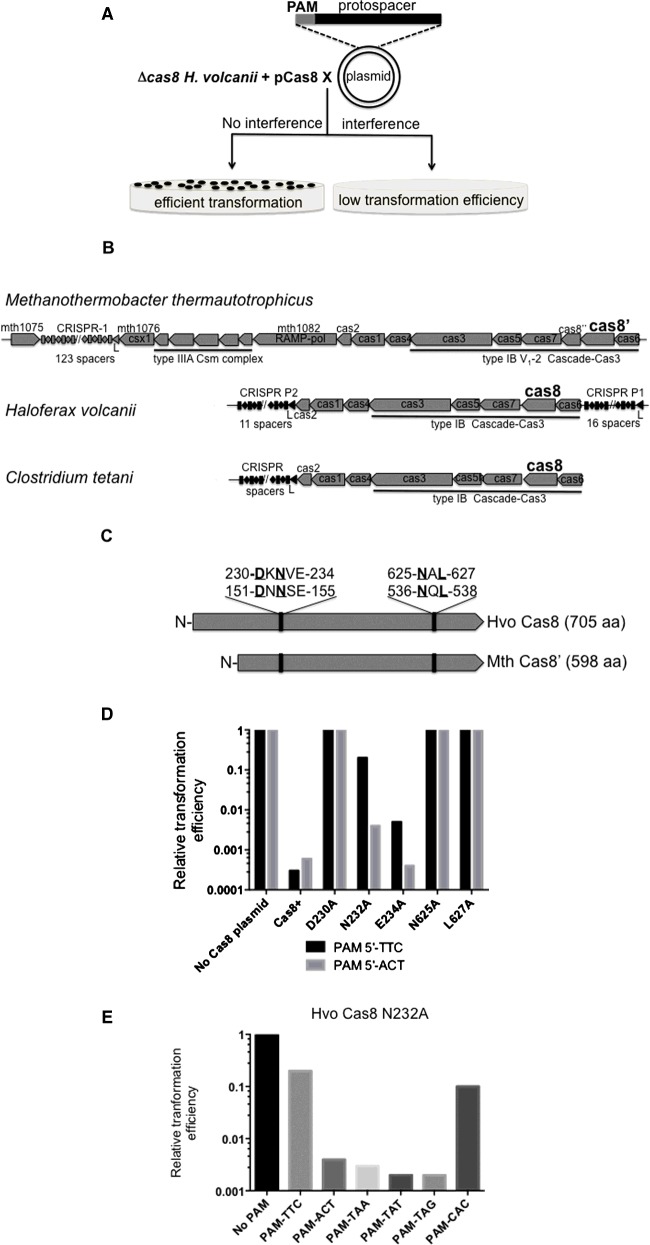
Effect on CRISPR interference of mutations in Archaeal Cas8 (**A**) Summary of *H. volcanii* (Hvo) plasmid protection assays. See also parts (**D**) and (**E**). Transformation efficiency was measured for a plasmid containing PAMs into Hvo Δ*cas8* cells complemented by pCas8^+^ or pCas8^MUTANT^ to determine their effects on interference. Efficient transformation of plasmid containing a PAM [43] and protospacer indicated loss of interference, manifest as colony growth supported by *leu* and *ura* complementation from the plasmid. (**B**) Type IB CRISPR-Cas systems of the euryarchaea *H. volcanii* (Hvo) and *M. thermautotrophicus* (Mth) and the bacterium *Clostridium tetani* (Cte). All have conserved gene order Cas8-Cas7-Cas5-Cas3. Mth contains an additional open reading frame (mth1089) called Cas8″ leading to its classification as a CRISPR type IB variant V_1_-2 and renaming of Cas8b [6] to Cas8′ [18]. The predicted amino acid sequence of Cas8″ shows no significant homology to any protein in database searches. (**C**) Cartoon illustrating two conserved amino acid patches in Hvo Cas8 and Mth Cas8′ homologues, which were targeted for mutagenesis in data presented in parts (**D**) and (**E**) and in the subsequent data Figures. A full alignment of Cas8/Cas8′/Cas8b is given in Supplementary Figure S1. (**D**) Relative interference efficiencies supported by Hvo Cas8 and its mutant proteins when measured in cells deleted for *cas8* (Δ*cas8*) in the assay summarized in Figure 1(A) using two different Hvo PAMs, as indicated. A transformation efficiency of 1.0 corresponds to no significant interference from an absence of Cas8 plasmid (‘no Cas8 plasmid’) or from mutations D230A, N625A and L627A. (**E**) Interference efficiency of Δ*cas8* Hvo cells expressing Cas8 N232A in the presence of plasmid harbouring any one of the six Hvo PAMs, as indicated.

### Cas8′ binding to DNA and R-loop nucleic acid substrates

As noted in Figure 1, homologues of Hvo Cas8 are present in other archaea and bacteria. Hvo is an extreme halophile, creating problems for analysis in native conditions of Hvo Cas8 binding to DNA/RNA substrates in EMSAs. Therefore, we purified the Cas8 homologue from *M. thermautotrophicus* (Mth) (Supplementary Figure S2), an organism amenable for analysis of its DNA-binding proteins. Mth Cas8 is called Cas8′ [18] because it is 119 amino acids shorter than Hvo Cas8, but it has the conserved amino acid residues required for CRISPR interference by Hvo Cas8, summarized in Figures 1C–1E. We constructed nucleic acid substrates for Cas8′ binding that centred on duplex DNA or R-loop shown in Figure 2(A). Substrates were either+or − PAM and contained a 5′-crRNA handle known to be important for interference in type IB CRISPR systems, whereas the 3′-handle is dispensable for interference in the same systems [52]. To predict Mth PAM, we analysed 123 spacers in Mth CRISPR-1, identifying protospacers from seven mobile genetic elements to deduce a PAM of 5′-CCN-3′, detailed in Supplementary Results and Supplementary Figure S3. CC dinucleotide PAM for Mth had been identified in a previous analysis [53], although reported as GG from the reverse complement of the Mth genome. Therefore we incorporated 5′-CCC into substrates for +PAM or 5′-AAA for −PAM.

**Figure 2:**
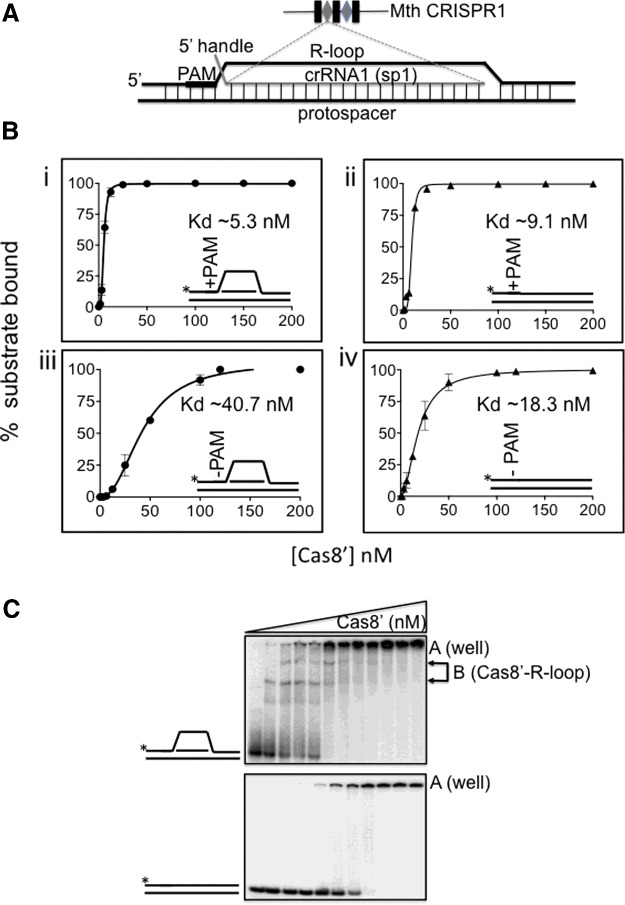
EMSAs for binding Cas8′ to duplex and R-loop with+and −PAM (**A**) Cartoon of R-loop/duplex model substrates used; sequences of DNA and RNA strands are given in supplemental data. +PAM 5′-CCN (CCC) or −PAM (AAA) sequences and a 5′ RNA handle were incorporated into duplex or R-loop as indicated. For R-loop, crRNA oligonucleotide (shown in red) was synthesized with sequence complementary to a spacer from Mth CRISPR-1. (**B**). Graphs comparing measurements of Cas8′ binding to duplex and R-loop substrates ± PAM, as indicated in each panel i–iv. Asterisks denote the ^32^P-end labelled DNA strand. EMSAs were in triplicate for plotting as mean values with bars for standard error. Substrate (2.0 nM) was mixed with Cas8′ at concentrations (nM): 0, 1.56, 3.125, 6.25, 12.5, 25.0, 50.0, 100, 120, 200. (**C**) Cas8′ gave distinct in gel complex when binding to R-loop (labelled B), compared with in well aggregates of protein–nucleic acid (complex A) with a duplex DNA.

Results of Cas8′ EMSAs are in Figures 2(B) and 2(C). Cas8′ bound to a +PAM R-loop or duplex DNA with highest affinity (Figure 2B respectively, *K*_d_ 5.3±0.7 nM and *K*_d_ 9.1 ±0.2 nM), compared with the same substrates −PAM (Figure 2B respectively, *K*_d_ 40.7±1.0 nM and *K*_d_ 18.3±0.8 nM). It was significant that Cas8′ bound to R-loops,+or −PAM, as distinct in-gel protein–DNA complexes, compared with in well aggregates of protein–DNA observed for Cas8′ mixed with duplex DNA (Figure 2C). These EMSAs suggested that Cas8′ in isolation can recognize PAM sequence and may have structural preference for binding stably to branched DNA or R-loops compared with duplex DNA.

### Physical interaction of Cas8′ with Cas5–Cas7 and PAM-dependent stimulation of nucleic acid binding

Cas5 and Cas7 are integral to bacterial and archaeal Cascades [9,19] and are predicted to function with Cas8 during CRISPR interference. Previous studies identified a Cas5–Cas7 complex in Hvo [22] and physical association of Cas8′ with Cas7 during fractionation of *Methanothermobacter* cell biomass [54]. In the present work, FLAG-tagged Cas7 was expressed in Hvo cells to detect protein interactors, identifying Cas8 when FLAG–Cas7 affinity enriched cell extracts were analysed by MS (Figure 3A). To test for physical interaction of Cas8′ with Cas5–Cas7, we first co-purified Mth Cas7 with Cas5 (Supplementary Figure S4A), the latter an N-terminal fusion to *E. coli* MBP, giving soluble MBP–Cas5–Cas7 that bound crRNA1. MBP–Cas5–Cas7 interacted physically with Cas8′ (Figure 3B), with the same results observed either with or without pre-incubation of Cas5–Cas7 with crRNA1. Cas8′ bound to MBP–Cas5–Cas7 pre-incubated with amylose resin (Figure 3B, top panel, lane 8), but did not bind to control amylose resin in BSA (Figure 3B, top panel, lane 6). MBP–Cas5–Cas7 was detected, as expected, in Figure 3(B), lane 8, but not in lane 6 containing BSA. The reciprocal reaction (MBP–Cas5–Cas7 to Ni^2+^-NTA bound Cas8′) was not effective because MBP–Cas5–Cas7 bound Ni^2+^-NTA even when Cas8′ was absent. These assays indicated physical interaction of Cas8′ with Cas5–Cas7, although a maximum of only 10% of Cas8′ input could be detected as bound to MBP–Cas5–Cas7 in these conditions.

**Figure 3:**
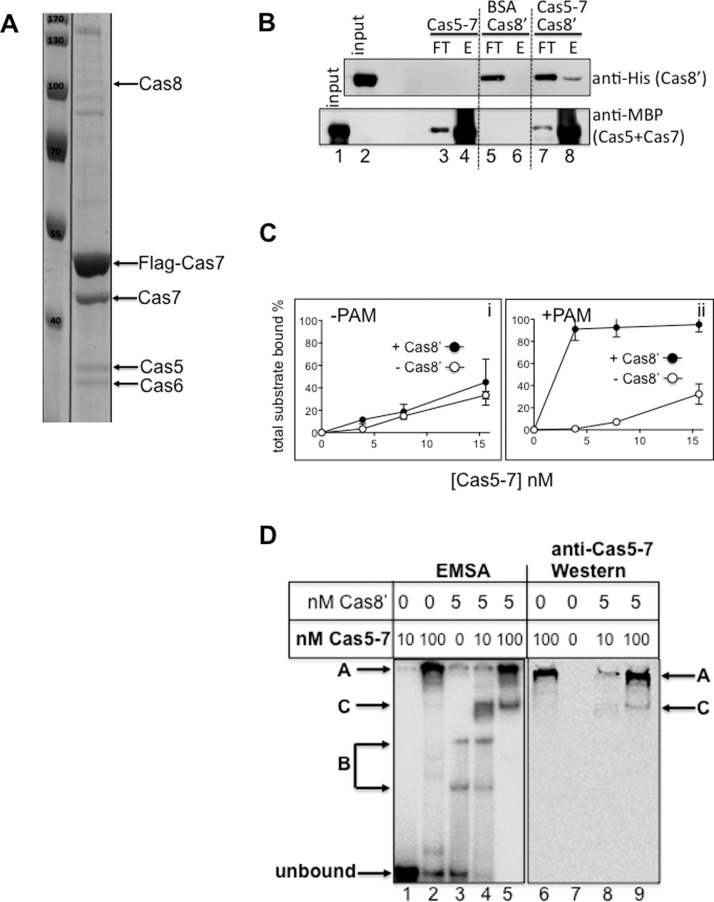
Interaction of Cas8 and Cas8′ with Cas5–Cas7 (**A**) Coomassie stained SDS/PAGE profile of co-purifying proteins with Flag-Tagged Cas7 expressed in *Haloferax* cells. Cas8 was detected by MS. (**B**) Reconstitution of physical interaction between purified *Methanothermobacter* Cas8′ (20 μg) with purified complex of affinity tagged *Methanothermobacter* Cas5–Cas7 (20 μg). Upper panel shows western blot using anti-(His)_6_ antibody to detect (His)_6_Cas8′ and the lower panel used anti-MBP antibody to detect MBP in MBP–Cas5–Cas7. ‘Input’ is a duplicate loading of total amount of used Cas8′ (upper panel) or Cas5–Cas7 (lower panel). Cas8′ was detected in the elution (**E**) after binding to amylose—MBP–Cas5–Cas7 (lane 8) but did not bind to amylose pre-bound with BSA (lane 6). (**C**) Measurements of duplex DNA binding ±PAM by *Methanothermobacter* Cas5–Cas7 either with or without Cas8′, as labelled. Data values for total DNA binding were calculated for each concentration of Cas5–Cas7 (4, 8, 15 nM) ±Cas8′ (5 nM). (**D**) Corresponding EMSA and western blots for detection of Cas5–Cas7 in a Cas8′ dependent in-gel complex. Lanes 1–5 (left panel) show phosphorimaged EMSA complexes arising from reactions binding of Cas5–Cas7 (complex A) or Cas8′ (B complexes). A new complex C was observed when Cas5–Cas7 and Cas8′ are present. Western blotting detected Cas5–Cas7 in complex C (lanes 8 and 9), as well as complex A (lanes 6, 8 and 9).

Cas8′ was tested for any influence effect on binding by Cas5–Cas7 to duplex ± PAM in EMSAs. Cas8′ stimulated total substrate binding when +PAM but had little effect on binding to −PAM (Figure 3C and representative gels in Supplementary Figure S4B). Significantly, EMSAs mixing Cas5–Cas7+Cas8′ showed a novel complex that was not present when either Cas5–Cas7 or Cas8 were alone (Figure 3D): Cas5–Cas7 at 10 nM or 100 nM formed in-well protein–DNA aggregates with, respectively, 8% and 67% of substrate (labelled A, Figure 3D, lanes 1 and 2). Cas8′ alone (5 nM) bound 55% of the substrate in distinct complexes (labelled B in Figure 3D, lane 3). A new complex (complex C) was defined when pre-mixing Cas8′ (5 nM) with Cas5–Cas7 (10 or 100 nM) and 90%–100% of substrate was bound (Figure 3D, lanes 4 and 5). Western blotting of identical EMSA using antibodies against MBP identified MBP–Cas5–Cas7 in complex C (Figure 3D, lanes 8 and 9), confirming that Cas5–Cas7 can form a distinct complex in EMSAs that is not an aggregate but dependent on Cas8′.

### Cas8 amino acid residues that are essential for interference *in vivo* are also essential for a Cas8′ nuclease activity *in vitro*

Mutant Mth Cas8′ proteins D151G, N153A and N536A were purified (Supplementary Figure S2), corresponding to mutations in Hvo Cas8 that had reduced or abolished interference activity in plasmid protection assays (Asp^230^, Asn^232^ and Asn^625^ Table 1A). Cas8′ mutant proteins were proficient in binding to R-loop +PAM (Supplementary Figure S5) and other substrates (result not shown) and interacted with MBP–Cas5-Cas7 (Supplementary Figure S6). We investigated N153A substrate binding in more detail because the corresponding Hvo mutation (Asn^232^) caused reduced interference to PAMs 5′-CAC or -TTC (Table 1B). Duplex and R-loop substrates containing PAM 5′-CCC were not bound by N153A appreciably better than substrates −PAM in EMSAs (Figure 4A), contrasting with the binding behaviour of wild-type Cas8′ (Figure 2). It is possible that this observation might account for subtly reduced interference, discussed later.

**Figure 4:**
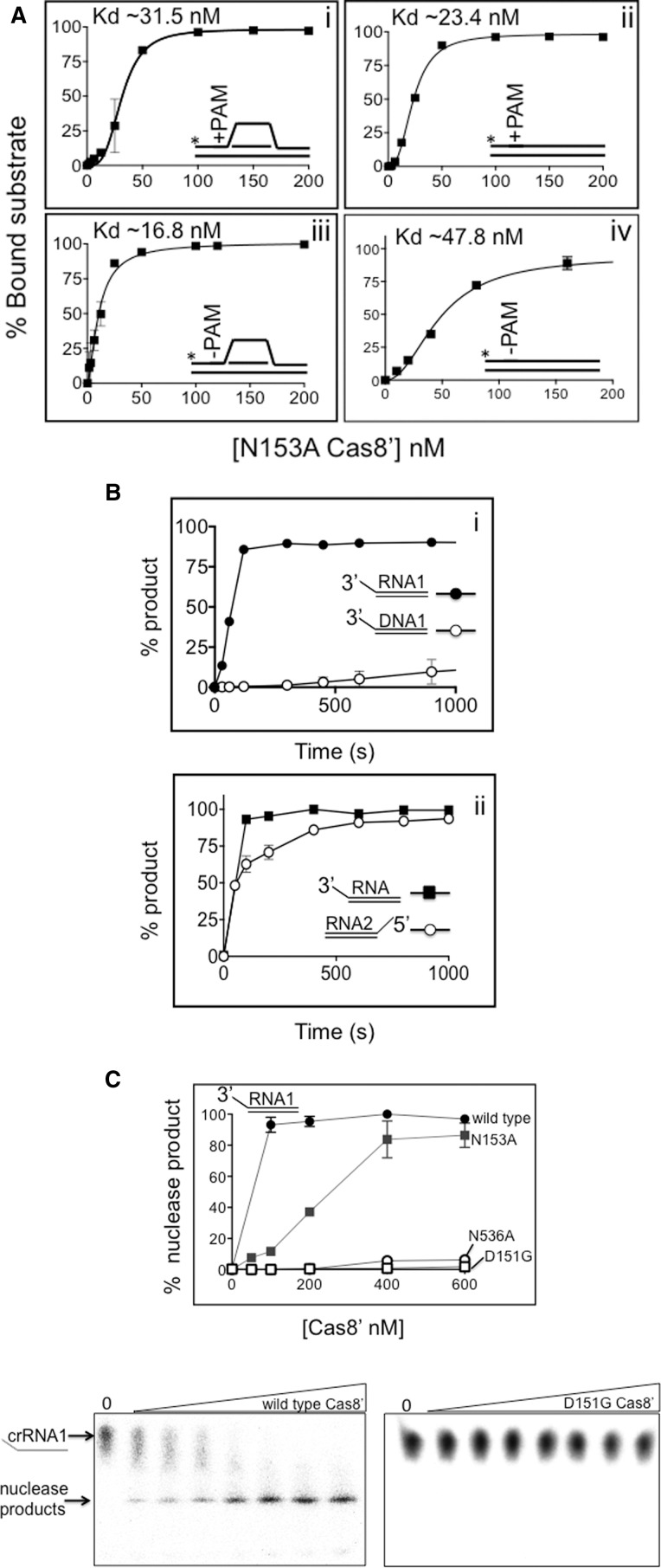
Cas8′ substrate binding and nuclease activities (**A**) Binding of N153A Cas8′ to substrates ±PAM as indicated in panels i–iv, for comparison with wild-type Cas8′ data in Figure 2(B). Asterisks denote the ^32^P-end labelled DNA strand. EMSAs were in triplicate for plotting as mean values with bars for standard error. Substrate (2 nM) was mixed with Cas8′ N153A at concentrations of (nM); 0, 1.56, 3.125, 6.25, 12.5, 25.0, 50.0, 100, 150 and 200 for panels i and ii; 0, 1.56, 3.125, 6.25, 12.5, 25.0, 50.0, 100, 120 and 200 for panel iii and 0, 6.25, 12.5, 20.0, 40.0, 80.0 and 160 nM in panel iv. (**B**) Graphs comparing Cas8′ (100 nM) nuclease activity as a function of time on; panel i, comparing DNase to RNase activity on 3′ ssDNA or ssRNA flaps (1 nM of each), panel ii, comparing RNase activity on 3′ or 5′ ssRNA flaps (1 nM of each). In each case the strand labelled was that with the single stranded region and assays were analysed on denaturing gels in triplicate for plotting as mean values with bars for standard error. (**C**) Comparative RNase activity of Cas8′ and mutants on 5′-ssRNA flap (1 nM) containing protein at 0, 50, 100, 200, 400 and 600 nM. In each panel the data points are means of assays in duplicate showing standard error and representative gels for wild-type Cas8′ and a catalytically inactive Cas8′ (D151G) are shown below the graph.

To test for Cas8′ catalytic activity correlating to lack of interference from the aspartic acid/asparagine mutants we re-visited previous work that had identified Mth Cas8′ nuclease activity targeting ssDNA flaps [54]. We now compared this DNase activity to equivalent RNase activity. Cas8′ was much more efficient as a nuclease, measured as a function of time, on an ssRNA flap of the same sequence as ssDNA (Figure 4Bi). In these reactions, RNA was present in a RNA–DNA hybrid, but RNA nuclease activity was not detected in RNA–RNA duplex, even though Cas8′ binding to DNA–DNA, RNA–DNA and RNA–RNA substrates was similar (Supplementary Figure S7). Cas8′ nuclease activity was detected on RNA with 3′- and 5′-ends (Figure 4Aii), but only on the strand with ssRNA overhang. Cas8′ D151G and N536A had negligible RNase activity (Figure 4C, with further examples of nuclease assay gels in Supplementary Figure S8A), but binding was intact (Supplementary Figure S8B). N153A had intermediate nuclease activity (Figure 4C). The catalytic activity of Cas8′ and inactivating mutations in conserved residues that are required for interference in Hvo, is evidence that Cas8′ is an RNA nuclease in cells, discussed more below.

## DISCUSSION

Cas8 proteins are candidates for guiding Cascade to invader DNA in some type I CRISPR systems [18,21]. Mutation of Cas8 had been implicated in loss of interference in *Haloferax* (Hvo) [43] and we investigated this using genetic analysis of Hvo Cas8 in CRISPR interference and nucleic acid binding and processing by Cas8′ from *Methanothermobacter* (Mth). We propose that Cas8/Cas8′ is part of Cascade, contributing to PAM and structure specific nucleic acid binding, influencing interaction of Cas5–Cas7 with nucleic acids. An interesting ssRNA nuclease activity of Cas8′ was detected *in vitro*, requiring conserved amino acids that are essential for interference *in vivo*.

Three lines of evidence indicated nucleic acid binding and PAM sensing by Cas8′, in isolation and when mixed with Cas5–Cas7. Firstly, isolated Cas8′ formed distinct complexes with R-loop substrates in EMSAs and predicted PAM 5′-CCN stimulated its binding to duplex and R-loop substrates (Figure 2). Second, the subtle PAM induced behaviour of Cas8 or Cas8′ mutated in respectively, Asn^232^ (Tables 1A and 1B) or Asn^153^ (Figure 4A) supports interaction with PAM either alone or when bound with Cas5–Cas7 in Cascade, an interaction that is perturbed by the asparagine mutation. Third, we observed enhanced substrate binding from Cas8′–Cas5–Cas7 +PAM, compared with either Cas5–Cas7 or Cas8′ alone, which was not observed when −PAM (Figure 3). Cas8′ in these assays converted Cas5–Cas7 protein aggregates into a distinct binding complex suggesting that Cas8′ modulates how Cas5–Cas7 can precisely assemble on the substrate, thereby controlling its aggregation. Based on *E. coli* Cascade structures detailing precise positioning for CasA relative to CasD (Cas5) [37–39], it is likely that interaction of Cas8 with Cas5–Cas7 is important for PAM sensing and for the choreography of Cascade binding to nucleic acids that leads to stable R-loop formation.

Mutation of Hvo Asp^230^ and Asn^625^ abolished Cas8 interference (Table 1A) and mutation of the corresponding residues in Mth Cas8′, Asp^151^ and Asn^536^, abolished ssRNA nuclease activity (Figure 4). Cas8′ degraded ssRNA with either 3′- or 5′-ends. However, we were unable to detect RNase activity *in vitro* from Hvo Cas8, despite using a wide range of high and low salt assay conditions, possibly because of instability of the purified protein that appeared during storage. Therefore we cannot conclude that abolished nuclease activity of Cas8′ D151G and N536A mutants explains the loss of genetic interference by Hvo Cas8 D230A and N625A mutants, but the correlation is interesting. Northern blotting for crRNA in Hvo Δ*cas8* cells showed that Cas8 is not needed for processing crRNA into pre-crRNA or crRNA in cells (Supplementary Figure S9). Also, Hvo cells do contain a nuclease that removes nts from the 3′-end of crRNA after processing from pre-crRNA but this RNase function is not altered if cells lack Cas8 (Anita Marchfelder, personal communication). 5′-crRNA handles are essential for interference in Hvo cells and are therefore not processed after crRNA formation [52]. Therefore the role of Cas8 RNase activity, if any, in Cascade-mediated CRISPR interference is undetermined. We cannot exclude the possibility that Cas8 RNase activity may be needed for some other aspect of RNA metabolism and processing in these organisms that has an indirectly important role for some type I CRISPR systems.

## Abbreviations

ACBB: amylose column-binding buffer
Cas: CRISPR-associated
Cascade: CRISPR-associated complex for antiviral defence
CRISPR: clustered regularly interspaced short palindromic repeat
crRNA: CRISPR RNA
His_6_–Cas8′: N-terminal hexahistidine-tagged Mth Cas8′
MBP: maltose-binding protein
PAM: protospacer adjacent motif
TBE: Tris-Borate-EDTA
Trp: tryptophan
